# The paradox of verbal autopsy in cause of death assignment: symptom question unreliability but predictive accuracy

**DOI:** 10.1186/s12963-016-0104-2

**Published:** 2016-10-18

**Authors:** Peter Serina, Ian Riley, Bernardo Hernandez, Abraham D. Flaxman, Devarsetty Praveen, Veronica Tallo, Rohina Joshi, Diozele Sanvictores, Andrea Stewart, Meghan D. Mooney, Christopher J. L. Murray, Alan D. Lopez

**Affiliations:** 1 Institute for Health Metrics and Evaluation, University of Washington, Seattle, WA USA; 2 School of Public Health, University of Queensland, Brisbane, Australia; 3 The George Institute for Global Health, Hyderabad, India; 4 The George Institute for Global Health, University of Sydney, Level 10, King George V Building 83-117 Missenden Rd, PO Box M201, Camperdown, 2050 NSW Australia; 5 Research Institute for Tropical Medicine, Muntinlupa City, Philippines; 6 Melbourne School of Population and Global Health, The University of Melbourne, Building 379, 207 Bouverie St, Carlton, 3053 VIC Australia

**Keywords:** Verbal autopsy, Cause of death, Reliability, Validity

## Abstract

**Background:**

We believe that it is important that governments understand the reliability of the mortality data which they have at their disposable to guide policy debates. In many instances, verbal autopsy (VA) will be the only source of mortality data for populations, yet little is known about how the accuracy of VA diagnoses is affected by the reliability of the symptom responses. We previously described the effect of the duration of time between death and VA administration on VA validity. In this paper, using the same dataset, we assess the relationship between the reliability and completeness of symptom responses and the reliability and accuracy of cause of death (COD) prediction.

**Methods:**

The study was based on VAs in the Population Health Metrics Research Consortium (PHMRC) VA Validation Dataset from study sites in Bohol and Manila, Philippines and Andhra Pradesh, India. The initial interview was repeated within 3–52 months of death. Question responses were assessed for reliability and completeness between the two survey rounds. COD was predicted by Tariff Method.

**Results:**

A sample of 4226 VAs was collected for 2113 decedents, including 1394 adults, 349 children, and 370 neonates. Mean question reliability was unexpectedly low (*kappa* = 0.447): 42.5 % of responses positive at the first interview were negative at the second, and 47.9 % of responses positive at the second had been negative at the first. Question reliability was greater for the short form of the PHMRC instrument (*kappa* = 0.497) and when analyzed at the level of the individual decedent (*kappa* = 0.610). Reliability at the level of the individual decedent was associated with COD predictive reliability and predictive accuracy.

**Conclusions:**

Families give coherent accounts of events leading to death but the details vary from interview to interview for the same case. Accounts are accurate but inconsistent; different subsets of symptoms are identified on each occasion. However, there are sufficient accurate and consistent subsets of symptoms to enable the Tariff Method to assign a COD.

Questions which contributed most to COD prediction were also the most reliable and consistent across repeat interviews; these have been included in the short form VA questionnaire. Accuracy and reliability of diagnosis for an individual death depend on the quality of interview. This has considerable implications for the progressive roll out of VAs into civil registration and vital statistics (CRVS) systems.

**Electronic supplementary material:**

The online version of this article (doi:10.1186/s12963-016-0104-2) contains supplementary material, which is available to authorized users.

## Background

Good quality data about the distribution of cause of death (COD) in a population in the context of well-functioning civil registration and vital statistics (CRVS) systems is fundamental to good public health practice [1, 2]. Ideally, COD data are based on medical certification and registration of all deaths [3]. However, most countries, particularly resource-poor ones, lack adequate systems for the collection of such data [1, 4]. In the absence of comprehensive medical certification of deaths, the primary means available for collecting useful mortality data at the population level is verbal autopsy (VA). A VA is a formal account, usually obtained from the family of a decedent, of a terminal illness or of the events leading to death. The Verbal Autopsy Instrument (VAI) used to collect these data comprises both a structured questionnaire and an open-ended narrative. Two modern VAIs in use are those developed by the World Health Organization (WHO) [5] and the Population Health Metrics Research Consortium (PHMRC) [6]. VAs are increasingly being considered as part of routine surveillance of COD through CRVS systems [7] and, in consequence, a number of publications have addressed issues of validity – the ability of a VA to predict COD accurately [8–11].

In an earlier paper, we described the effect of the duration of time between death and VA administration (recall period) on VA validity. The analysis was based on a study of pairs of verbal autopsies for 2113 decedents collected at various time periods after death from field sites in Andhra Pradesh in India, and in Bohol and Manila in the Philippines. The data were entered into the PHMRC VAI and analysed using the Tariff Method [12]. Tariff is a simple additive algorithm that creates a score, or tariff, for each question/symptom pair in a VA and uses summed scores to assign COD [12, 13]; it is the recommended data-driven method developed from the PHMRC, based on a study of comparative diagnostic accuracy [8]. The probability of a correct COD assignment was shown to decrease by 0.55 % for each month after death that a VA was conducted [14].

We believe that it is important that governments understand the reliability of the mortality data which they have at their disposable to guide policy debates. In many instances, VA will be the only source of mortality data for populations, yet little is known about how the accuracy of VA diagnoses is affected by the reliability of the symptom responses. We are aware of only one publication which examines a small number of maternal deaths in Burkina Faso and Indonesia. Repeatability (i.e., reliability) was found to be moderate in interview material and lower in terms of individual deaths [15].

In this paper, based on the same dataset as described above, we assess the relationship between the consistency of individual symptom responses and the reliability and accuracy of COD prediction. We define *reliability* as “the degree to which the results obtained by a measurement can be replicated,” and *accuracy* as the ability of the VA “to correctly identify a person who did or did not have [die from] the disease of interest” [16].

## Methods

### Data

VAs for this study were collected for deaths occurring from 2007 to 2010 in Bohol, Manila, and Andhra Pradesh. VAs were administered in two separate rounds. The first round was collected between 6 days and 5 months after death as a part of the PHMRC Gold Standard Verbal Autopsy Validation Study (PHMRC study) [6]. In the second round, a subset of families was revisited and retest VAs conducted 3–20 months after death. A second wave of second-round VAs was collected under a grant from the Australian National Health & Medical Health Council (NHMRC Project Grant 631494) in Bohol only, 18–52 months after death (Bohol (2)). All data collection procedures were approved by the Internal Review Board of the University of Washington, Seattle, WA, USA; School of Public Health, University of Queensland; George Institute for Global Health, Hyderabad, India; and Research Institute for Tropical Medicine, Alabang, Metro Manila, Philippines. All information on VAs was collected after obtaining signed consent from the informants.

The general methodology of the PHMRC study has been described in detail elsewhere and is summarized here for convenience [6]. Gold standard (GS) clinical diagnostic criteria for hospital deaths were reported for a list of 34 adult, 21 child, and six neonatal causes including stillbirths that were mutually exclusive and collectively exhaustive (Additional file 1). Deaths with hospital records fulfilling the GS criteria were identified in each of the sites. Interviewers blinded to the GS diagnosis then gathered information about the events leading up to the decedent’s death using the PHMRC VAI. The PHMRC Data Base contains 12,535 verbal autopsies with GS diagnoses (7846 adults, 2064 children, 1620 neonates, and 1005 stillbirths). Retest VAs for the present study were collected following the PHMRC protocol [17]. Only decedents with a retest survey are included.

The PHMRC VAI includes both closed-ended questions and an open-ended narrative. Close-ended questions concern symptoms of the terminal illness, details of underlying disease conditions that had been obtained from health service providers, risk behaviors (tobacco and alcohol), and details of interaction with health services. Questions collected either as continuous or categorical variables were transformed into dichotomous variables which we refer to as question items. Text items, also dichotomous variables, were derived from an open-ended narrative using a text mining procedure that identifies keywords and word groups [12].

The VAI was applied in two lengths: long form and short form. The long form is the original PHMRC VAI which was used in this study [6]. The short form was developed for use on hand-held electronic tablets to routinely administer VAs in civil registration systems. It contains those questions in the long form which contribute most to the accurate prediction of COD as assessed by formal item reduction methods [18]. The total number of questions was reduced from 459 (long-form VAI) to 245 (short-form VAI); the number of questions in the adult module was reduced from 183 to 113, in the child module from 127 to 72, and in the neonate module from 149 to 69.

### VA COD assignment

Data collected for this study were analysed using Tariff Method [12, 13]. The Tariff Method is based on the strength of the relationship between individual symptoms and individual causes of death. Each symptom is assigned a *tariff score* for each COD. The tariff score depends on the strength of association between a single symptom and a particular COD and on its distribution across all causes. In effect, the tariff score normalizes the symptom distribution across causes. Prediction of COD in an individual decedent is based on a summation of tariff scores for that death. COD lists by module (age group) are shown in Additional file 1. An individual death from a particular cause is likely to be associated with a subset of those symptoms but not the full set, i.e., different individual deaths are likely to be associated with different subsets of symptoms.

The tariff scores for a symptom strongly associated with a particular COD will have high standard deviations, and tariff scores for symptoms more common across many different CODs will have low standard deviations. The short form of the PHMRC VAI was created by first ranking all question items in the long form and then successively deleting low-ranking symptoms, simultaneously observing the effect of deletions on the performance characteristics (COD predictive accuracy) of the instrument using formal item-reduction methods [18]. The short form thus contains only those questions which make the greatest contribution to predictive accuracy.

The Tariff Method has been shown to have a high level of validity at both the individual and population levels when compared with other methods of VA analysis [8]. Because nearly all measures of performance of a VA method for assigning COD vary as a function of the true cause of death composition in the study population [19], the Tariff Method was developed using 500 train-test data analysis datasets, each with a different COD composition, created by sampling the entire PHMRC GS dataset [13]. Data for the present study were analyzed using Tariff 2.0, a revised version of the Tariff Method [12]. The Tariff 2.0 method was retrained to exclude the 2113 deaths which were the subject of this study to maintain out of sample predictive validity. It should be noted that Tariff 2.0 makes a prediction of “indeterminate cause of death” when the model lacks sufficient information to assign a COD.

VA performance in assigning COD to an individual decedent was assessed using chance-corrected concordance (CCC) which measures sensitivity adjusted for chance [19]. The CCC is the mean of cause-specific chance-corrected concordances calculated from the 500 train-test datasets, and so does not vary with cause composition. The overall effect of including an indeterminate category, which was treated as a separate cause of death in this analysis, is to reduce apparent CCC by removing potential sources of compensating error.

### Data analysis

#### Measures of performance

The data set was structured in the form of a matrix. Each cell of the matrix contains two numbers which represent responses to a question which has been asked twice (Table 1). The rows represent responses to individual questions. The columns represent responses to questions about individual decedents. The extent of agreement between Rounds One and Two can be shown in two-by-two tables (Table 2) which sum the results for rows and columns separately and provide the basis for the agreement metrics shown in Table 3. From these we derive the reliability measures which are described below.

**Table 1:** Structure of the data set

		Individuals by Round											
		A		B		C		D		E		F	
		One	Two	One	Two	One	Two	One	Two	One	Two	One	Two
Quest-ions	One	0	0	1	0	0	1	1	0	1	1	1	0
	Two	0	0	1	1	0	1	0	0	1	0	1	1
	Three	0	1	0	0	1	1	0	1	0	0	0	1
	Four	1	0	1	1	1	0	0	0	1	1	0	0

**Table 2:** Agreement of a single column or row in Table 9

		Round One		
		Yes (1)	No (0)	Total
Round Two	Yes (1)	a	b	m1
	No (0)	c	d	m2
	Total	n1	n2	N

**Table 3:** Definitions of metrics referred to in this paper

Term	Definition	Formula
Question endorsement ratio	The proportion of “yes” responses for a given question.	$\frac{1}{2}*\ \left(\frac{\left( Yes\ survey\ 1\right)}{Total\ \#\ questions} + \frac{\left( Yes\ survey\ 2\right)}{Total\ \#\ questions}\right)$
Question proportion agreement*	The proportion of questions for which first and second verbal autopsy survey rounds were consistent (p_0_)	${p}_0=\frac{\#\ \left( Yes\ survey\ 1,\ Yes\ survey\ 2\right) + \#\ \left( No\ survey\ 2,\ No\ survey\ 1\right)}{Total\ \#\ questions}$
Question kappa	Proportion question agreement (p_0_) adjusted by proportion expected agreement (p_e_) for first and second survey rounds	$\mathrm{kappa}=\frac{p_o-{p}_e}{1-{p}_e}$
Question proportion gain	The proportion of questions with a “no” response in the first survey round reported as a “yes” response in the second survey round.	$Proportion\ Gain = \frac{\left( No\ survey\ 1,\kern0.5em Yes\ survey\ 2\right)}{Total\ \#\ Yes\kern0.5em survey\kern0.5em 2}$
Question proportion loss	The proportion of questions with a “yes” response in the first survey round reported as a “no” response in the second survey round.	$Proportion\ Loss=\frac{\#\ \left( Yes\ survey\;1, No\ survey\kern0.5em 2\right)}{Total\ \#\ Yes\ survey\ 1}$
Decedent proportion agreement	Proportion agreement for all question responses for a given decedent	${p}_0=\frac{\#\ \left( Yes\ survey\ 1, Yes\ survey\ 2\right)+\#\left( No\ survey\ 2, No\ survey\;1\right)}{Total\ \#\ questions}$
Decedent question kappa	Kappa for question response for a given decedent	
COD proportion agreement	Proportion agreement for COD predictions for all decedents	${p}_0=\frac{{\displaystyle \sum \left( COD\ j\ survey\ 1, COD\ j\ survey\ 2\right)}}{Total\ \#\ decedents}$
COD kappa	Kappa for COD predictions for a given decedent	
Cause-specific proportion agreement	Proportion agreement for COD predictions for a given COD	${p}_{0,j}=\frac{{\displaystyle \sum \left(\left( COD\ j\ survey\ 1, COD\ j\ survey\ 2\right)+\left( Not\ COD\ j\ survey\ 1, Not\ COD\ j\ survey\ 2\right)\right)}}{Total\ \#\ questions}$
Cause-specific kappa	Kappa for COD predictions for a given COD	
Prediction match	Binary indicator. If predicted COD was the same for first and second VAs, if prediction match = 1; if not the same, prediction match = 0.	
Chance-corrected concordance (CCC)	The sensitivity of a cause of death estimate adjusted for chance.	$CC{C}_j=\frac{\left(\frac{T{P}_j}{T{P}_j+F{N}_j}\right)-\left(\frac{1}{N}\right)}{1-\left(\frac{1}{N}\right)}$ where TP_j_ is true positives or number of decedents with gold standard cause j correctly assigned to cause j, FN is false negatives or the number of decedents incorrectly assigned to cause j, and N is the number of causes analyzed. TP plus FN equals the true number of deaths due cause j.

#### Endorsement ratio

An endorsed question is one to which the respondent answered “Yes.” The endorsement ratio is the proportion of questions that have been endorsed.

#### Question reliability at the level of the question

The two common measures of reliability are proportion agreement (the proportion of questions with the same “Yes” and “No” answers at two interviews) and *kappa*, which adjusts proportion agreement for chance. Proportion agreement has been included here for the sake of completeness. Tariff Method makes use only of endorsed questions. When, as in this study, endorsement ratios are low, proportion agreement puts undue emphasis on negative responses. Two other metrics examine the reliability of positive responses; a) the proportion of questions endorsed in the first round of VA that were not endorsed in the second round, and b) the proportion of questions not endorsed at the first round that were endorsed at the second round. We refer to these as *question proportion loss* and *question proportion gain,* respectively*.*

#### Question reliability at the level of the decedent

Decedent question *kappa* [20] measures agreement between the first and second rounds of verbal autopsy for all question responses about a given decedent. It is fundamental to the measurement of the effect of contextual factors, such as change of respondent or of interviewer, and on reliability of responses in individuals, as well as to prediction of COD.

#### Reliability of COD prediction at the level of the individual

To quantify the reliability of individual COD assignments, a dichotomous variable, labeled “*prediction match*,” measures whether predictions of the COD were the same at the first and second round of interviews.

### Regression analysis of the relationship between question reliability, COD reliability, and the context of the interview

#### Effects of contextual factors on question reliability (decedent question kappa)

##### Regression 1

Using linear regression, we examined the effects on question reliability of changes between survey rounds in the respondent or in the interviewer, of time between survey rounds, and of module and site. Because of co-linearity, Bohol (1) and Bohol (2) were combined into a single reference group.

*decedent question kappa = β*_*0*_ *+ β*_*1*_ *respondent match + β*_*2*_ *interviewer match + β*_*3*_ *recall period 6–11 months + β*_*4*_ *recall period 12–23 months + β*_*5*_ *recall period ≥ 24 months + β*_*6*_ *module + β*_*7*_ *site*

#### Effect of question reliability on COD prediction reliability

##### Regression 2

We then examined the effects of question reliability on COD prediction reliability using logistic regression. Because each individual death in the data set has two VAs, a correct assignment was significantly more likely in the second VA if it had also been selected in the first VA (correlation coefficient of 0.485). We therefore relaxed the assumption of independence between observations for verbal autopsy diagnoses from the same decedent. Setting a fixed effect that differentiated between first and second round VAs would detract from measuring the effect of the true predictor of interest: COD prediction match. We employed a clustered sandwich variance estimator [21] using the cluster option in Stata for each regression, which relaxes the assumption of independence of two VAs from the one decedent.

Decedent question kappa is the independent variable in the regression. Because kappa is bound between zero and one, we multiplied decedent question kappa by ten to make odds ratios more intuitively understandable.

*P(prediction match) = logit(β*_*0*_ *+ β*_*1*_ (decedent question kappa*10))

## Results

A convenience sample of 4226 VAs was collected for 2113 decedents (Table 4). Details of more adult deaths were collected (1394 decedents) than were details of child (349) or neonatal deaths (370). The average period between death and VA interview was 1.84 months. More than half of second-round VAs (1067) were collected within 6–11 months of the first VA (Table 5); 13.4 % was collected within 5 months of death, 50.5 % within 6–11 months, 14.3 % within 12–23 months, and 21.8 % at a period >23 months.

**Table 4:** Number of decedents by site and module and survey dates by site

	Bohol (1)	Bohol (2)	Manila	Andhra Pradesh	Total
Adult	235	312	190	657	1394
Child	45	42	59	203	349
Neonate	69	107	37	157	370
Total	349	461	286	1017	2113
Survey Dates Round 1	6 Jan 2009–30 Jan 2010	30 Jul 2007–24 Jul 2008	8 Jan 2009–30 Mar 2010	1 May 2009–30 Apr 2010	30 Jul 2007–30 Apr 2010
Survey Dates Round 2	1 Mar 2010–28 Jul 2010	23 Nov 2010–13 Oct 2011	3 Mar 2010–30 Jul 2010	18 Feb 2010–16 Aug 2010	18 Feb 2010–13 Oct 2011

**Table 5:** Number of verbal autopsies by time between VA interviews

Time between VA interviews	Andhra Pradesh	Manila	Bohol (1)	Bohol (2)	Total
0–5 months	174	70	39	0	283
6–11 months	824	127	116	0	1067
12–23 months	19	89	194	0	302
≥24 months	0	0	0	461	461
Total	1017	286	349	461	2113

### Measures of reliability

Table 6 shows means and confidence intervals for the reliability measures as applied to the full length VAI. It includes question items but not text items. The mean question endorsement ratio for all modules was 0.177 (0.156, 0.197). The mean endorsement ratio for adults of 0.143 (0.119, 0.167) was much lower than that for children of 0.223 (0.169, 0.276) or that for neonates of 0.194 (0.153, 0.235). Mean question proportion agreement for all modules was 0.898 (0.896, 0.899), reflecting the high proportion of negative responses. Mean question kappa was 0.447 (0.421, 0.474). The mean question kappa for adults of 0.398 (0.363, 0.433) was much lower than that for children of 0.495 (0.434, 0.556) or that for neonates of 0.488 (0.437, 0.539). Mean proportion loss of positive responses in the first round was 0.425. The mean proportion loss for adults of 0.481 (0.446, 0.516) was much higher than that for children of 0.344 (0.287, 0.401) or that for neonates of 0.399 (0.347, 0.452). Mean question proportion gain of positive responses in the second round was 0.479 (0.451, 0.506). Mean proportion gain was much higher for adults of 0.537 (0.501, 0.573) than that for children of 0.410 (0.350, 0.469) or that for neonates of 0.442 (0.387, 0.497). Mean decedent question kappa was 0.610 (0.603, 0.617). COD prediction match was 0.474 (0.452, 0.495). COD was correctly assigned in 41.2 % (39.7–42.7 %) of cases.

**Table 6:** Mean and confidence interval measures of reliability for question responses and COD predictions in the long form of the Verbal Autopsy Instrument

Full length PHMRC VAI					
		Adult	Child	Neonate	Overall
Question reliability	Question endorsement ratio	0.143 (0.119, 0.167)	0.223 (0.169, 0.276)	0.194 (0.153, 0.235)	0.177 (0.156, 0.197)
	Question proportion agreement	0.893 (0.892, 0.895)	0.900 (0.897, 0.903)	0.918 (0.916, 0.921)	0.898 (0.896, 0.899)
	Question kappa	0.398 (0.363, 0.433)	0.495 (0.434, 0.556)	0.488 (0.437, 0.539)	0.447 (0.421, 0.474)
	Question proportion loss	0.481 (0.446, 0.516)	0.344 (0.287, 0.401)	0.399 (0.347, 0.452)	0.425 (0.398, 0.451)
	Question proportion gain	0.537 (0.501, 0.573)	0.410 (0.350, 0.469)	0.442 (0.387, 0.497)	0.479 (0.451, 0.506)
Decedent question reliability	Decedent proportion agreement	0.893 (0.892, 0.895)	0.900 (0.897, 0.903)	0.918 (0.916, 0.921)	0.898 (0.896, 0.899)
	Decedent question kappa	0.554 (0.547, 0.561)	0.706 (0.693, 0.720)	0.730 (0.716, 0.744)	0.610 (0.603, 0.617)
COD prediction reliability	COD prediction Match	0.412 (0.387, 0.438)	0.530 (0.477, 0.583)	0.651 (0.603, 0.700)	0.474 (0.452, 0.495)
COD prediction validity	Correct assignment of COD	0.379 (0.361, 0.397)	0.362 (0.327, 0.398)	0.581 (0.545, 0.617)	0.412 (0.397, 0.427)

Endorsement ratios for text items were, in general, less than for question items. A table showing reliability metrics for both question and text items, i.e., for the instrument as a whole, is to be found in Additional file 2. The difference in prediction match (0.535 versus 0.474) and correct assignment of COD (49.1 % of cases versus 41.2 % of cases) between the two tables reflects the additional contribution that text items make to diagnosis.

Table 7 shows the same metrics when applied to the short form of the PHMRC VAI which contains those questions which have the greatest predictive accuracy. The mean question endorsement ratio of 0.178 (0.153, 0.203) was similar to the endorsement ratio in the long form. Other question reliability metrics have improved. Mean question kappa in the short form of 0.497 (0.464, 0.530) was much higher than that in the long form of 0.447 (0.421, 0.474). Question proportion loss of 0.388 (0.355, 0.421) and question proportion gain of 0.441 (0.407, 0.476) were lower in the short form. Mean decedent question kappa of 0.676 (0.670, 0.683) was significantly higher in the short form. COD prediction reliability and COD prediction validity were at the same level in long and short forms, confirming that the questions deleted from the long form contributed little, if anything, to the prediction of COD. In other words, questions that contributed most to the prediction of COD were also the most reliable, as defined earlier.

**Table 7:** Mean and confidence interval measures of reliability for question responses and COD predictions in the short form of the Verbal Autopsy Instrument

Short form of the VAI					
		Adult	Child	Neonate	Overall
Question reliability	Question endorsement ratio	0.117 (0.089, 0.146)	0.224 (0.172, 0.275)	0.249 (0.193, 0.306)	0.178 (0.153, 0.203)
	Question proportion agreement	0.927 (0.926, 0.928)	0.896 (0.892, 0.900)	0.913 (0.909, 0.916)	0.922 (0.921, 0.923)
	Question kappa	0.438 (0.394, 0.483)	0.570 (0.503, 0.637)	0.539 (0.471, 0.607)	0.497 (0.464, 0.530)
	Question proportion loss	0.457 (0.413, 0.501)	0.297 (0.237, 0.356)	0.347 (0.276, 0.419)	0.388 (0.355, 0.421)
	Question proportion gain	0.517 (0.470, 0.563)	0.359 (0.297, 0.422)	0.376 (0.304, 0.448)	0.441 (0.407, 0.476)
Decedent question reliability	Decedent proportion agreement	0.927 (0.926, 0.928)	0.896 (0.892, 0.900)	0.913 (0.909, 0.916)	0.922 (0.921, 0.923)
	Decedent question kappa	0.642 (0.634, 0.650)	0.727 (0.715, 0.740)	0.757 (0.741, 0.772)	0.676 (0.670, 0.683)
COD prediction reliability	COD prediction Match	0.415 (0.389, 0.441)	0.524 (0.472, 0.577)	0.719 (0.673, 0.765)	0.487 (0.465, 0.508)
COD prediction validity	Correct assignment of COD	0.376 (0.358, 0.394)	0.360 (0.324, 0.395)	0.618 (0.582, 0.653)	0.416 (0.401, 0.430)

### Regression equations: examination of the relationship between question reliability, the context of the interview, and COD reliability

#### Effect of contextual factors on question reliability at the level of the individual (decedent question kappa)

Decedent question kappa is the dependent variable in Regression 1, a linear regression, which uses contextual factors as independent variables. Table 8 shows that if the respondent was the same at the first and second interviews, reliability increased by 0.062; if the interviewer was the same, reliability increased by 0.029. A period between interviews of >6 months had a small effect on reliability (< −0.02) but there was no evidence of decreasing reliability after 6 months. The largest effects were by survey module (0.149 increase in reliability with the child module and 0.160 increase with the neonatal module.) Reliability was greater at the Andhra Pradesh (0.021) and Manila field sites (0.28). The regression “explained” 30.1 % of the variation in decedent question kappa.

**Table 8:** Linear regression of the effect of contextual factors on decedent question kappa (Regression 1)

	Coefficient (95 % CI)
Respondent Match	0.062 (0.047, 0.077)
Interviewer Match	0.029 (0.011, 0.047)
0–6 months between VAs (reference)	
6–11 months between VAs	−0.017 (−0.033, −0.002)
12–23 months between VAs	−0.019 (−0.040, 0.002)
≥24 months between Vas	−0.015 (−0.037, 0.007)
Adult (reference)	
Child	0.149 (0.135, 0.162)
Neonate	0.160 (0.146, 0.173)
Andhra Pradesh	0.021 (0.003, 0.039)
Bohol (1) (reference)	
Bohol (2) (reference)	
Manila	0.028 (0.009, 0.048)
R-squared	0.301

#### Relationship between reliability at the individual level and reliability of COD

Prediction match was the dependent variable in Regression 2, a logistic regression. The odds ratio for the independent variable (decedent question kappa)*10 was 1.421 (1.330, 1.519). This can be interpreted as meaning that an increase of 0.1 in mean decedent question kappa would make COD prediction match between first- and second-round VAs 42.1 % more likely.

#### Relationship between COD prediction reliability and validity

Estimates of COD prediction reliability and validity in Table 9 are based on both text and question items. The table shows that, at the individual level, COD was correctly assigned to 49.1 % of deaths. (There was a small difference in correct assignment between the first survey round (50.5 %) and the second survey round (47.7 %)). The table also shows that if the prediction matched between the two survey rounds, then 68.1 % of predictions were correct, whereas if predictions did not match, then only 27.2 % were correct.

**Table 9:** Relationship between COD prediction match and the correct assignment of COD

	Prediction match						
		Yes		No		Total	
		*N*	%	*N*	%	*N*	%
Correct assignment	Yes	1540	68.1 %	535	27.2 %	2076	49.1 %
	No	720	31.9 %	1431	72.8 %	2151	50.9 %
	Total	2260	100.0 %	1966	100.0 %	4227	100.0 %

## Discussion

What benchmarks are there for the levels of predictive accuracy described in this study? The two major sources for COD statistics in resource-poor countries are medical certificates of COD for hospital deaths and VA for non-hospital deaths. Table 10 shows CCC for COD assigned by Tariff 2.0 to VAs in the first round of this study with all VAs in PHMRC dataset [12]. It compares these with CCC of death certificates written in 34 public hospitals in Mexico. The hospital deaths were based on gold standard cases, i.e., on cases selected because the clinical records were of sufficient quality to provide a firm basis for the diagnosis. It shows CCC for cases where the true underlying cause of death (UCOD) was assigned correctly as well as for cases where the true UCOD appeared anywhere in the death certificate. The first of these reflects the actual performance of hospital physicians in writing death certificates; the second reflects the maximum information that could be extracted from the death certificates by well-trained coders. This is the only study available to make such a direct comparison [22].

**Table 10:** Comparison of Chance Corrected Concordance (CCC) for VAs with CCC for death certificates from 34 Mexican public hospitals

Module	Tariff 2.0 Reliability dataset^a^		Tariff 2.0 full dataset		Selected Mexican hospitals			
	UCOD	95 % CI	UCOD	95 % CI	UCOD	95 % CI	All diagnoses	95 % CI
Adult	0.491	(0.490, 0.493)	0.505	(0.502, 0.507)	0.665	(65.9, 66.9)	0.759	(75.4, 76.3)
Child	0.474	(0.470, 0.477)	0.525	(0.521, 0.530)	0.385	(37.0, 40.0)	0.640	(61.4, 66.3)
Neonate	0.445	(0.442, 0.448)	0.451	(0.446, 0.454)	0.543	(52.2, 55.6)	0.589	(56.9, 60.5)

^a^ First Round VA’s with both question and text items

Table 10 shows that CCC for deaths in the first-round VAs in this study was comparable with CCC for the PHMRC study as a whole. CCC for COD from VAs was 9–16 % less than CCC for UCOD in hospital death and 11–25 % less if the UCOD appeared anywhere in the death certificate. The careful selection of gold standard cases for the Mexican hospital study means that results represent an ideal rather than established practice. A recent systematic review of hospital COD statistics concluded that misdiagnosis in medical certificates of COD was the norm rather than the exception [23]. The primary motivation for introducing VAs into CRVS systems is to provide population-level mortality statistics – i.e., Cause Specific Mortality Fractions (CSMFs), and not to provide COD for individuals. There is no direct relationship between CCC and the accuracy of prediction of CSMFs. However, when Tariff 2.0 data is compared with the Mexican hospital statistics the accuracy of CSMF prediction for VAs was in the range 77–83 % and for death certificates in the range 82–89 %, i.e., there was less difference between the two than might have been expected.

This study was initially designed to determine the effect of the duration of time since death on VA symptom recall [14]. Our aims, when planning this analysis of question reliability, were to establish levels of reliability and to examine the effects of contextual factors. We assumed question reliability to be a pre-requisite for internal validity: i.e., for the reliability and accuracy of COD predictions. Instead we found paradoxically low levels of question reliability in conjunction with levels of COD predictive accuracy consistent with other VA studies [8, 13].

There are four key attributes of VA question responses: 1) reliability, 2) completeness, 3) the contribution the symptom response makes to diagnosis, (i.e., to predictive accuracy), and 4) the accuracy with which responses reflect the real-life experience of the terminal illness. The last attribute could not be measured in the current database. Note also that although reliability and completeness are related, responses to a question can be reliable but not complete, and *vice versa*.

We make a number of observations. First, levels of question reliability were unexpectedly low (Table 6). Mean question kappa for all modules was 0.447; question kappa for the adult module was 0.398.A kappa value >0.75 is generally accepted to reflect excellent agreement beyond chance; a value <0.4 represents poor agreement; and a value in the range 0.4–0.75 represents fair to good agreement [24]. Question reliability in the long form of the VAI was fair at best.

Second, the structured VAI was introduced in the first place to ensure completeness of question responses. Yet 42.5 % of positive responses at the first interview were negative at the second and 47.9 % of positive responses at the second had been negative at the first. By this measure alone, the subset of responses obtained at each of the interviews was incomplete.

On the other hand, the measures of question reliability and completeness improved when applied to the short form of the VAI which contained the questions which contributed most to the accuracy of prediction as measured by Tariff (Table 7). We conclude that question responses for those symptoms which contribute most to diagnosis are more reliable and more complete than for those symptoms which make little or no contribution.

Differences between the long and short forms were preserved when the data were analyzed at the level of the individual. Mean decedent question kappa was 0.610 in the long form and 0.676 in the short form. In comparing question kappa with decedent question kappa we interpret kappa not only as a measure of agreement beyond chance but as an intraclass correlation coefficient [24]. In other words, question reliability is not only a characteristic of individual questions; it is, even more importantly, a characteristic of responses to questions about individual decedents.

Contextual factors, operating at the level of individual decedents, explained 30 % of the variation in decedent question kappa (Regression 1). Variation between modules was responsible for approximately 15–16 % of the variation; non-matching of respondents was responsible for 6 % and of interviewers for 3 %. These are not large effects. It should be remembered, however, that the study was conducted in a research environment and much care had been taken in the selection of respondents and in the training of interviewers.

A hierarchy of effects of reliability/non-reliability has now been established. An increase of 0.1 in mean decedent question kappa would make a COD prediction match between the first and second round VAs 42.1 % more likely (Regression 2). COD prediction match, in turn, was strongly associated with the accuracy of prediction of COD (Table 7).

Reliability and completeness are not only attributes of question responses *per se*, they are also attributes of responses to questions about individual deaths. VAs of deaths in infants and small children conform more closely to the clinical encounter than do VAs in adults. The former rely on caretaker observations of clinical signs, whereas the latter are a mix of observations of signs and of secondhand accounts of conversations about symptoms. The level of detail in the communication between caretaker and decedent will have depended in part on their personalities and in part on the nature of their relationship. A woman, for example, might give quite different accounts of menstrual irregularity to her sister and to her husband.

We conclude from the foregoing remarks that a VA, based as it is on the recollections of family members weeks or months after the event, is not and cannot be as accurate as a hospital diagnosis based not only on a clinical history but also on clinical examination and investigation. However, at the population level, automated VA is a very useful and cost-effective approach to determining the cause composition of mortality.

Two further issues deserve consideration in seeking to answer the question of why Tariff Method is as accurate as it is. The first relates to the characteristics of the open-ended narrative and the second to the nature of Tariff Method itself.

At the end of interview, in the long form of the PHMRC VAI, the respondent is asked, “Could you please summarize, or tell us in your own words, any additional information about the illness and/or death of your loved one?” This is referred to as the “open narrative.” The text items referred to in the preceding sections were extracted from this narrative. It was noted that the endorsement ratio for text items was lower than for question items, i.e., responses in the open-ended narrative were less complete and less reliable than in the questionnaire. The tariff score for a symptom question mentioned spontaneously was frequently higher than when elicited through the questionnaire [18]. This is to treat the open narrative simply as a source of information for the construction of a symptom list; however, the open narrative is much more than this. It is a coherent account of a series of events, which incorporates interpretations of those events. As such, it is most likely a more accurate reflection of stored memories than are responses to a questionnaire. Before responding to a question, the informant may be considering issues such as sensitivity (how severe was the symptom?) and relevance (was this symptom part of a terminal illness or of something else?), and so on.

## Conclusions

It was noted earlier that each COD is associated with a set of symptoms. The full set of symptoms as recorded for the hospital gold standard cases could be regarded as the ideal for a particular COD. The individual death from a particular cause is likely to be associated with a subset of those symptoms but not the full set, and different individual deaths are likely to be associated with different subsets. Caretakers were reporting aspects of the terminal illness. Accuracy in assigning COD is dependent on the reliability of the prediction but is not so dependent on the reliability of responses to symptom questions.

The paradox of low levels of reliability and completeness in response to symptom questions in association with high levels of predictive accuracy of VAs (given inherent technical limitations) is, in our view, resolved. We conclude that although informants may report different aspects of the same illness on different occasions – that is, their reports may be unreliable and incomplete – they still reflect a sufficient number of symptoms sufficiently accurately for the Tariff Method to make an accurate diagnosis. This finding adds further support to the basic philosophy of the short-form VA questionnaire, namely that only items are being retained that are robust and have sufficient discriminatory power for major causes of death.

Question reliability at the level of the individual – decedent question kappa – is an important factor in COD prediction reliability and hence of COD predictive accuracy. It follows that predictive accuracy is dependent on the quality of interview – a most important conclusion to keep in mind as VAs progressively move from a research environment to routine CRVS systems.

## Additional files

Additional file 1: Cause list for PHMRC VA study by module. (DOCX 13 kb)
Additional file 2: Mean and confidence interval measures of reliability for question responses and COD predictions including and excluding text in the short and long forms of the Verbal Autopsy Instrument. (DOCX 18 kb)

## Abbreviations

CCC: Chance corrected concordance
COD: Cause of death
CRVS: Civil registration and vital statistics systems
GS: Gold standard
NHMRC: Australian National Health and Medical Research Council
PHMRC: Population Health Metrics Research Consortium
UCOD: Underlying cause of death
VA: Verbal autopsy
VAI: Verbal autopsy instrument
WHO: World Health Organization
