# Supplementary material for: The paradox of verbal autopsy in cause of death assignment: symptom question unreliability but predictive accuracy
Source: Popul Health Metr. 2016 Oct 18;14:41. doi: 10.1186/s12963-016-0104-2 (PMC5101673; doi:10.1186/s12963-016-0104-2)
Supplement: Additional file 2: — Mean and confidence interval measures of reliability for question responses and COD predictions including and excluding text in the short and long forms of the Verbal Autopsy Instrument. (DOCX 18 kb) [file 12963_2016_104_MOESM2_ESM.docx]

| **Additional file 2.**  **Mean and confidence interval measures of reliability for question responses and COD predictions including text in the short and long forms of the Verbal Autopsy Instrument** | | | | | | | | |  |  | |  |
| --- | --- | --- | --- | --- | --- | --- | --- | --- | --- | --- | --- | --- |
| Short form of the VAI | | | | | | Full length PHMRC VAI | | | | | | |
|  |  | Adult | Child | Neonate | Overall |  |  | Adult | Child | | Neonate | Overall |
| Question reliability | Question endorsement rate | 0.115 (0.088, 0.141) | 0.203 (0.157, 0.250) | 0.231 (0.178, 0.284) | 0.168 (0.145, 0.191) | Question reliability | Question endorsement rate | 0.090 (0.076, 0.104) | 0.166 (0.129, 0.203) | | 0.169 (0.136, 0.201) | 0.125 (0.111, 0.138) |
|  | Question proportion agreement | 0.926 (0.912, 0.940) | 0.897 (0.873, 0.921) | 0.907 (0.890, 0.925) | 0.913 (0.903, 0.924) |  | Question proportion agreement | 0.923 (0.914, 0.933) | 0.907 (0.891, 0.923) | | 0.909 (0.895, 0.922) | 0.916 (0.909, 0.923) |
|  | Question kappa | 0.441 (0.399, 0.482) | 0.549 (0.489, 0.610) | 0.525 (0.462, 0.588) | 0.491 (0.460, 0.521) |  | Question kappa | 0.355 (0.332, 0.378) | 0.436 (0.391, 0.481) | | 0.440 (0.395, 0.485) | 0.392 (0.373, 0.412) |
|  | Proportion loss | 0.453 (0.412, 0.494) | 0.313 (0.258, 0.367) | 0.356 (0.290, 0.422) | 0.391 (0.361, 0.421) |  | Proportion loss | 0.537 (0.513, 0.562) | 0.425 (0.378, 0.472) | | 0.441 (0.393, 0.488) | 0.490 (0.470, 0.511) |
|  | Proportion gain | 0.518 (0.475, 0.562) | 0.391 (0.332, 0.450) | 0.401 (0.333, 0.468) | 0.454 (0.423, 0.486) |  | Proportion gain | 0.618 (0.593, 0.642) | 0.492 (0.445, 0.539) | | 0.501 (0.453, 0.548) | 0.563 (0.543, 0.584) |
| Decedent question reliability | Decedent proportion agreement | 0.950 (0.950, 0.950) | 0.886 (0.886, 0.886) | 0.913 (0.912, 0.913) | 0.933 (0.932, 0.934) | Decedent question reliability | Decedent proportion agreement | 0.954 (0.954, 0.954) | 0.917 (0.917, 0.917) | | 0.943 (0.943, 0.944) | 0.946 (0.945, 0.947) |
|  | Decedent kappa | 0.634 (0.626, 0.642) | 0.725 (0.713, 0.738) | 0.748 (0.732, 0.764) | 0.669 (0.663, 0.676) |  | Decedent kappa | 0.522 (0.516, 0.529) | 0.671 (0.659, 0.683) | | 0.679 (0.666, 0.692) | 0.574 (0.568, 0.580) |
| COD prediction reliability | Prediction Match | 0.489 (0.463, 0.516) | 0.544 (0.492, 0.597) | 0.719 (0.673, 0.765) | 0.539 (0.517, 0.560) | COD prediction reliability | Prediction Match | 0.492 (0.466, 0.518) | 0.564 (0.512, 0.617) | | 0.668 (0.619, 0.716) | 0.535 (0.514, 0.556) |
| COD prediction validity | Correct assignment | 0.468 (0.449, 0.486) | 0.453 (0.416, 0.490) | 0.618 (0.582, 0.653) | 0.491 (0.476, 0.507) | COD prediction validity | Correct assignment | 0.470 (0.451, 0.488) | 0.451 (0.414, 0.488) | | 0.609 (0.574, 0.645) | 0.491 (0.476, 0.506) |
|  |  |  |  |  |  |  |  |  |  | |  |  |
| **Mean and confidence interval measures of reliability for question responses and COD predictions excluding text in the short and long forms of the Verbal Autopsy Instrument** | | | | | | | | | | | | |
| Short form of the VAI | | | | | | Full length PHMRC VAI | | | | | | |
|  |  | Adult | Child | Neonate | Overall |  |  | Adult | Child | | Neonate | Overall |
| Question reliability | Question endorsement rate | 0.117 (0.089, 0.146) | 0.224 (0.172, 0.275) | 0.249 (0.193, 0.306) | 0.178 (0.153, 0.203) | Question reliability | Question endorsement rate | 0.143 (0.119, 0.167) | 0.223 (0.169, 0.276) | | 0.194 (0.153, 0.235) | 0.177 (0.156, 0.197) |
|  | Question proportion agreement | 0.926 (0.911, 0.941) | 0.889 (0.862, 0.917) | 0.906 (0.887, 0.924) | 0.912 (0.901, 0.923) |  | Question proportion agreement | 0.892 (0.876, 0.908) | 0.896 (0.874, 0.918) | | 0.913 (0.897, 0.928) | 0.899 (0.889, 0.909) |
|  | Question kappa | 0.438 (0.394, 0.483) | 0.570 (0.503, 0.637) | 0.539 (0.471, 0.607) | 0.497 (0.464, 0.530) |  | Question kappa | 0.398 (0.363, 0.433) | 0.495 (0.434, 0.556) | | 0.488 (0.437, 0.539) | 0.447 (0.421, 0.474) |
|  | Proportion loss | 0.457 (0.413, 0.501) | 0.297 (0.237, 0.356) | 0.347 (0.276, 0.419) | 0.388 (0.355, 0.421) |  | Proportion loss | 0.481 (0.446, 0.516) | 0.344 (0.287, 0.401) | | 0.399 (0.347, 0.452) | 0.425 (0.398, 0.451) |
|  | Proportion gain | 0.517 (0.470, 0.563) | 0.359 (0.297, 0.422) | 0.376 (0.304, 0.448) | 0.441 (0.407, 0.476) |  | Proportion gain | 0.537 (0.501, 0.573) | 0.410 (0.350, 0.469) | | 0.442 (0.387, 0.497) | 0.479 (0.451, 0.506) |
| Decedent question reliability | Decedent proportion agreement | 0.947 (0.947, 0.947) | 0.866 (0.866, 0.867) | 0.905 (0.904, 0.905) | 0.926 (0.925, 0.928) | Decedent question reliability | Decedent proportion agreement | 0.938 (0.938, 0.938) | 0.891 (0.890, 0.891) | | 0.934 (0.934, 0.935) | 0.930 (0.929, 0.930) |
|  | Decedent kappa | 0.642 (0.634, 0.650) | 0.727 (0.715, 0.740) | 0.757 (0.741, 0.772) | 0.676 (0.670, 0.683) |  | Decedent kappa | 0.554 (0.547, 0.561) | 0.706 (0.693, 0.720) | | 0.730 (0.716, 0.744) | 0.610 (0.603, 0.617) |
| COD prediction reliability | Prediction Match | 0.415 (0.389, 0.441) | 0.524 (0.472, 0.577) | 0.719 (0.673, 0.765) | 0.487 (0.465, 0.508) | COD prediction reliability | Prediction Match | 0.412 (0.387, 0.438) | 0.530 (0.477, 0.583) | | 0.651 (0.603, 0.700) | 0.474 (0.452, 0.495) |
